# Supplementary material for: Analysis of zebrafish periderm enhancers facilitates identification of a regulatory variant near human KRT8/18
Source: eLife. 2020 Feb 7;9:e51325. doi: 10.7554/eLife.51325 (PMC7039683; doi:10.7554/eLife.51325)
Supplement: Supplementary file 2. — (a) Alignments summary for enhancer homology test between ppl-10 and PPL-8.3. (b) Alignments details for enhancer homology test between ppl-10 and PPL-8.3. All alignments were conducted using the CLUSTALW algorithm with default parameters via the Clustal Omega server (https://www.ebi.ac.uk/Tools/msa/clustalo/). Alignments were then annotated to highlight identical blocks of length 5 to 6 bp long (cyan) or longer (yellow). See Materials and methods for further details on the choice of enhancer fragments used in these alignments. [file elife-51325-supp2.docx]

**a Summary of ClustalO alignment between zebrafish *ppl*-10 enhancers and PPL-8.3**

| **Sequence Two (pairwise), or Sequence Three (3-way)** | **Length (bp)** | **# of identical positions in alignment (*)** | **# of null characters inserted (-)** | **% identity of human 400 bp** | **% identity, normalized by insertions** | **5-mers** | **6-mers** | **7-mers** | **8-mers** | **9-mers** | **10-mers** | **11-mers** |
| --- | --- | --- | --- | --- | --- | --- | --- | --- | --- | --- | --- | --- |
| Mouse plus strand (+) | 409 | 231 | 57 | 57.8% | 50.5% | 10 | 0 | 2 | 2 | 2 | 2 | 1 |
| Mouse minus strand (-) | 409 | 159 | 117 | 39.8% | 30.8% | 2 | 2 | 1 | 0 | 1 | 0 | 0 |
| Mouse reverse sequence | 409 | 167 | 85 | 41.8% | 34.4% | 2 | 0 | 0 | 0 | 0 | 0 | 0 |
| Mouse scrambled sequence 1 | 409 | 157 | 71 | 39.3% | 33.3% | 1 | 0 | 0 | 0 | 0 | 0 | 0 |
| Mouse scrambled sequence 2 | 409 | 163 | 81 | 40.8% | 33.9% | 3 | 1 | 0 | 0 | 0 | 0 | 0 |
| Mouse scrambled sequence 3 | 409 | 160 | 59 | 40.0% | 34.9% | 1 | 1 | 0 | 0 | 0 | 0 | 0 |
| Zebrafish plus strand (+) | 467 | 181 | 73 | 45.3% | 38.3% | 5 | 0 | 0 | 0 | 0 | 0 | 0 |
| Zebrafish minus strand (-) | 467 | 158 | 107 | 39.5% | 31.2% | 2 | 0 | 0 | 0 | 0 | 0 | 0 |
| Zebrafish reverse sequence | 467 | 168 | 107 | 42.0% | 33.1% | 2 | 0 | 1 | 0 | 0 | 0 | 0 |
| Zebrafish scrambled sequence 1 | 467 | 168 | 95 | 42.0% | 33.9% | 4 | 1 | 0 | 0 | 0 | 0 | 0 |
| Zebrafish scrambled sequence 2 | 467 | 166 | 135 | 41.5% | 31.0% | 2 | 1 | 0 | 0 | 0 | 0 | 0 |
| Zebrafish scrambled sequence 3 | 467 | 174 | 73 | 43.5% | 36.8% | 1 | 2 | 1 | 0 | 0 | 0 | 0 |
| 3-way w/ zf plus strand (+) | 467 | 96 | 158 | 24.0% | 17.2% | 0 | 0 | 0 | 0 | 0 | 0 | 0 |
| 3-way w/ zf minus strand (-) | 467 | 97 | 227 | 24.3% | 15.5% | 0 | 0 | 0 | 0 | 0 | 0 | 0 |
| 3-way w/ zf reverse sequence | 467 | 99 | 248 | 24.8% | 15.3% | 0 | 0 | 0 | 0 | 0 | 0 | 0 |
| 3-way w/ zf scrambled sequence 1 | 467 | 110 | 170 | 27.5% | 19.3% | 1 | 1 | 0 | 0 | 0 | 0 | 0 |
| 3-way w/ zf scrambled sequence 2 | 467 | 86 | 216 | 21.5% | 14.0% | 0 | 0 | 0 | 0 | 0 | 0 | 0 |
| 3-way w/ zf scrambled sequence 3 | 467 | 90 | 140 | 22.5% | 16.7% | 0 | 0 | 0 | 0 | 0 | 0 | 0 |

**b Details for ClustalO alignment between zebrafish *ppl*-10 enhancers and PPL-8.3**

**Alignment #1.** Shown below is the pair-wise alignment of the core human *periplakin* (*ppl*) enhancer conserved in mammals (400 bp) aligned to the homologous 409 bp block in mouse. These sequences correspond to the plus strand (+) sequences located upstream of *ppl*, which is transcribed to the left in each genome. Alignment #1 serves as a positive control for the described enhancer homology tests.

**9-mer (a)**

Hu_400+ TTCTGACACAACCTGCTACACAT----CTTGGATCCCACTTGTCAAGCCTGGCCCTA**GGC** 56

Mm_409+ CTATCCCACAGACTGGTGACCCTGGAGCTCAGACACTCCACTGGAGAGCTATCCCTG**GGC** 60

* * **** *** * * * ** ** * * * ** **** *******

**10-mer (b) 10-mer (c)**

Hu_400+ **CTCTCA**GG------GGTGGGTACATCAAGTG**AGGAAGTCAC**ACTG**TCAGGGCAGA**AAGAG 110

Mm_409+ **CTCTCA**AATTCACCATGAGAGCTTCTCTGCA**AGGAAGTCAC**CCCA**TCAGGGCAGA**--GTG 118

********** * * ************** * ************** * *

**8-mer (d) 9-mer (e)** (f) (g) **7-mer (h)**

Hu_400+ GGGCTG**GGTGGGGC**CGG**ACAGGCTCT**GC-TGGCTCAGG**ATCCC**G**GCAGG**ACAGG**CTGCCC** 169

Mm_409+ GTTCTA**GGTGGGGC**TAA**ACAGGCTCT**ACCAGGCACAGT**ATCCC**T**GCAGG**GCAGA**CTGCCC** 178

* ** ************ ************* * *** *** ********* ********* *** **********

(i) (j)

Hu_400+ **A**ACCCAGCCCCAAAACTCCCATCTTCTGTCCAACGCCCAAG**CCTGC**TTCCTC**CCTGC**CCC 229

Mm_409+ **A**GCCTCCTGTTCACG--------------CCCATGCTTTTT**CCTGC**CTGCTG**CCTGC**TCC 224

***** ** * ** * ** ********* * ** ********* **

(k) **(l) (m)**

Hu_400+ ---------------ACCTCACCTCACACTGCC**CAGAC**TGGTGGGGCT**GCATT**T**TTGGG**G 274

Mm_409+ TCCCCTTCTGGGCCGTGCCAGGCTCATGCAGAG**CAGAC**CAGTACAGCC**GCATT**C**TTGGG**C 284

* **** * * ********* ** ** ********* *********

**7-mer (n)** **8-mer(o) 11-mer (p)**

Hu_400+ C-CGGTGCCA**GGTGTGA**TGGA-----GGACAGCC**CAGGTCAG**G**AGGTCAGGGCC**CCGTTT 328

Mm_409+ AAGTGTCTCT**GGTGTGA**ATGACCCTGGGGCTGGT**CAGGTCAG**A**AGGTCAGGGCC**TTGTTC 344

** * *********** ** ** * * ************ *************** ***

(q) (r)

Hu_400+ **CCTCC**CAAGCCAGGATGACTCAGTGATGGACAGACTAGGCCACAAGGGG-CC**GAACA**CAA 387

Mm_409+ **CCTCC**AGTGCCCAAGTGATA--------GATGAGTCAGGCTACAAAGGGGCA**GAACA**AGA 396

********* *** *** ** **** **** *** * ********* *

(s)

Hu_400+ AG**CCAAA**TTAGGG 400

Mm_409+ AA**CCAAA**CCGAGT 409

* ********* *

**Alignment #2.** Shown below is the pair-wise alignment of the core human *ppl* enhancer (Hu_400+) aligned to the reverse complement of the homologous 409 bp block in mouse (Mm_409-).

Hu_400+ ------------------**TTCTG**ACACAACCTGCTACACATCTTGGATCCCACTTGTCA- 41

Mm_409- ACTCGGTTTGGTTTCTTG**TTCTG**CCCCTTTGTAGCCTGACTCATCTATCACTTGGGCACT 60

********* * * * ** * *** * *

Hu_400+ ------AGCCT**GGCCCT**AGGCCTCTCAGGGGTGGGTACATCAAGTGAGGAAGTCACACTG 95

Mm_409- GGAGGGAACAA**GGCCCT**GACCTTCTGACCTG----------ACCAGCCCCAGGGTCATTC 110

* * ********** * *** * * * * ** ** *

**7-mer**

Hu_400+ TCAGGGCAGAAAGAGGGGCTGGGTGGGGCCGG-----ACAG**GCTCTGC**TGGCTCAGGATC 150

Mm_409- ACACCAGAGACACTTGCCCAAGAATGCGGCTGTACTGGTCT**GCTCTGC**ATGAGCCTGGCA 170

** *** * * * * * * * * *********** * * *

Hu_400+ CCGGCAGGACAGGCTGCC----CAACCCAGCCCCAAAACTC----------------CCA 190

Mm_409- CGGCCCAGAAGGGGAGGAGCAGGCAGCAGGCAGGAAAAAGCATGGGCGTGAACAGGAGGC 230

* * * ** ** * * * ** **** *

**9-mer**

Hu_400+ TCTTCTGTCCAACGCCC---------AA**GCCTG**CT----TCCTCCCT**GCCCCACCT**CACC 237

Mm_409- TGGGCAGTCTGCCCTGCAGGGATACTGT**GCCTG**GTAGAGCCTGTTTA**GCCCCACCT**AGAA 290

* * *** * * ********* * * *************

Hu_400+ TCACA**CTGCCC**AGACTGGTGGGGCTGCATTTTTGGGGCCGGTGCCAGGTGTGATGGAGGA 297

Mm_409- CCACT**CTGCCC**TGATGGGGTG-ACTTCCTTGCAGAGAA--------GCTCTCATGGTGAA 341

*** ********** ** ** * ** * ** * * * * * **** * *

Hu_400+ CAGCCCAGGTCAGGAGGTCAGGGCCCCGTTTCCTCCCAAGCCAGGATGACTCAGTGATGG 357

Mm_409- TTTGAGAGGCCCA-----------------------------GGGATAGCTCTCCAGTGG 372

*** * **** *** ***

Hu_400+ ACAGACTAGGCCACAAGGGGCCGAACACAAAGCCAAATTAGGG 400

Mm_409- AGTGTCTGAGCTCCAGGGTCACCAGTCTGTGG-GATAG----- 409

* * ** ** ** ** * * * * *

**Alignment #3.** Shown below is the pair-wise alignment of the core human *ppl* enhancer (Hu_400+) aligned to the reverse sequence of the homologous 409 bp block in mouse (Mm_409R). This alignment constitutes a negative control as the reverse sequence is a non-biological sequence.

Hu_400+ TTCTGACACAACCTGCTACACATCTTGGATCCCACTTGTCAAGCCTGGCCCTAGGCCTCT 60

Mm_409R ---TGAGCCAAACC----------AAAGAACAAGACGGGGAAACATCG------------ 35

*** *** * ** * * ** * * *

Hu_400+ CAGGGGTGGGTACATCAA---GTGAGGAAGTCACAC-TGTCAGGGCAGAAAGAG-GGGCT 115

Mm_409R ---GACTGAGTAGATAGTGAACCCGTGACCTCCCTTGTTCCGGGACTGGAAGACTGGACT 92

* ** *** ** ** ** * * * ** * * **** ** **

Hu_400+ GGGTGGGGCCGGACAGGCTCTGCTGGCTCAGGA--TCCCGGCAGGACAGGCTGCCCAACC 173

Mm_409R GGTCGGGGTCCCAGTAAGTGTGGTCTCTGTGAACGGGTTCTTACGCCGACATGACCAGAC 152

** **** * * * ** * ** * * * * * ** *** *

Hu_400+ CAGCCCC-----AAAACTCCCA-----TCTTCTGTCCAACGCCCAAGCCTGCTTCCTCCC 223

Mm_409R GAGACGTACTCGGACCGTGCCGGGTCTTCCCCTCCTCGTCCGTCGTCCGTCCTTTTTCGT 212

** * * * ** ** ** * * * * * *** **

Hu_400+ TGCCCCACCTCACCTCACACTGCC**CAGAC**TGGTG---------GGGCTGCATTTTTGGG- 273

Mm_409R ACCCGCACTTGTCCTCCGACCCGT**CAGAC**GGGACGTCCCTATGACACGGACCATCTCGGA 272

** *** * **** ** ********* ** * * * * **

Hu_400+ --------GCCGGTGCCAGGTGTGATGGAGGACAGCCCAGGTCAGGAGGTCAGGGCCCCG 325

Mm_409R CAAATCGGGGTGGATCTTGGTGAGACGGGACTACCCCACTGAAGGAACGTCTCTTCGAGA 332

* ** * **** ** ** ** * * * *** *

Hu_400+ TTTCCTCCCAAGC---CAGGATGACTCAGTGAT-------GG**ACAGA**--CTAGGCCACAA 373

Mm_409R GTACCACTTAAACTCTCCGGGTCCCTATCGAGAGGTCACCTC**ACAGA**CTCGAGGTCCCAG 392

* ** * ** * * ** * ** ********* * *** * **

Hu_400+ GGGGCCGAACACAAAGCCAAATTAGGG 400

Mm_409R TGGTCAGACACCCTAT---------C- 409

** * ** * *

**Alignments #’s 4, 5, and 6.** Shown below are three pair-wise alignments of the core human *ppl* enhancer (Hu_400+) aligned to one of three (S1, S2, and S3) different Fisher-Yates shuffled sequences of the homologous 409 bp plus-strand block in mouse (Mm_409+S1/S2/S3). These pair-wise alignments serve as negative controls.

Alignment #4

Hu_400+ TTCTGACACAACCTGCTACACAT**CTTGG**ATCCCACTTGTCAAGCCTGGCCCTAGGCCTCT 60

Mm_409+S1 --------AGAGCTC------CG**CTTGG**---CCTGTGCTCAAATG------------AAG 31

* ** ********* ** * ****

Hu_400+ CAGGGGTGGGTACATCAAGTGAGGAAGTCACAC--TGTCAGGGCAGAAAGAGGGGCTGGG 118

Mm_409+S1 CAGAGACCGGCCAAGCACAAGGGGCGTTTACAACTTGCTGCGGGAAGTAGAGCTTCTGAG 91

*** * ** * ** * ** * *** ** ** * **** *** *

Hu_400+ TGGGGCCGGACAGG---CTCTGCTGGCTCAGGATCCCGGCAGGACAGGCTGCCCAACCCA 175

Mm_409+S1 ACCGACCGGCCGGCACACCCTACTTGGCTTGAACCACCAATTTGTGGTGCACATGATCCT 151

* **** * * * ** ** * * * * * * * * **

Hu_400+ GCCCCAAAACTCCCATCTTCTGTCCAACGCCCAAGCCTGCTTCCTCCCTGCCCCACCT-- 233

Mm_409+S1 TCCGGACCGCACCAACGATTTGACGGAGTCCGCCTGCAGATTGGGGCCGAGCTGTTTTGG 211

** * * ** * * ** * * ** * * ** ** * *

Hu_400+ ----CACCTCACACTGCCCAGACTGG---------TGGGGCTGCATTTTTGGGGCCGGTG 280

Mm_409+S1 AACCGGAGTATAAGTCCTCTGTGTCTTCGTCTCTCAGTGGTTTCGTTATGT--CCCTCCG 269

* * * * * * * * ** * * ** * ** *

Hu_400+ CCAGGTGTGATGGAGGACAGCCC-------AGGTCAGGAGG---TCAGGGCCCCGTTTCC 330

Mm_409+S1 CCTCACGGGATCAATGGCACCGCATCCCCAAGCACGGGTGCGAAGACAGGCAGAGATTCT 329

** * *** * * ** * * ** * ** * *** * ***

Hu_400+ TCCCAAGCCAGGATGACTCAGTGATGGACAGACTAGGCCACAAGGGGCCGAACACAAAGC 390

Mm_409+S1 GGCCACGTCAATACTCCCCGTAACTCCACAGTACAGCGCTAATAGTGGGTAGTCCGCCCT 389

*** * ** * * * * **** ** * * * * * *

Hu_400+ CAAATTAGGG---------- 400

Mm_409+S1 ATACGGAGAGCCGCCCACCC 409

* ** *

Alignment #5

Hu_400+ ---TTCTGACACAACCTGCTACACATCT----------TGGATCCCACTTGTCAAGCCTG 47

Mm_409+S2 TAAACGAGGAGCTACCTCATACCGACCTAAGGGAGAATCGTCCCGCACCTGTAACCACA- 59

* * **** *** * ** * * *** *** * *

Hu_400+ GCCCTAGGCCTCTCAGGGGTGGGTACATCAAGTGAGGAAGTCACACTGTCAGGGCAGA-- 105

Mm_409+S2 ------TTCCTGATCGGTCTAGGGTCATTTCCTTCGTAGTCCTAACCACCACACCCGATT 113

*** ** * ** *** * * * * ** ** * **

Hu_400+ ---------------AAGAGGGGCTGGGTGGGGCCGGACAGGCTCT**GCTGG**CTCAGGATC 150

Mm_409+S2 GAAGACTGGCTCCCAACCGGGAGTTGGGCATGCCACG---GCCCTC**GCTGG**GCCTCCCTC 170

* ** * **** * * * * * ********* * **

Hu_400+ CCGGCAGGACAGGCTGCCCAACCCAGCCCCAA-AACTCCCATCTTCTGTCCAACGCCCAA 209

Mm_409+S2 CTGTGCGCCCGGAATTTGGAGTACGCCCACGGTACCACCCGGACGCGCTAGACAGGTAAG 230

* * * * * * * * ** * * * *** * * * * *

Hu_400+ GCCTGCTTCCTCCCTGCC**CCACC**TCACCTCACACTGCCCAGACTGGTGGGGCTGCATTTT 269

Mm_409+S2 GGATCCATTGAGTCCGGG**CCACC**GTGATGATTACTGGGCAGGAAACTCGGAGCGCAAGGG 290

* * * * * * ********* **** *** * ** ***

Hu_400+ T--GGGGCCGGTGCCAGGTGTGATGGAGGACAGCCCAGGTCAGGAG**GTCAG**GGCCCCGTT 327

Mm_409+S2 TGCGAAGCAGTTAACTGGTTTCT-----------------------**GTCAG**TAC--C-AT 324

* * ** * * * *** * ********* * * *

Hu_400+ TCCTC**CCAAGC**-CAGGATGACTCAGTGATGGACAGACTAGGCCACAAGGGGCCGAACACA 386

Mm_409+S2 TGTTG**CCAAGC**CAACTGTTACGCCTTGTGTGTGAGTCACCAGGGCATGTGCCAACTCAAT 384

* * ********** * * ** * ** * ** * ** * * * **

Hu_400+ AAGCCAAATTAGGG----------- 400

Mm_409+S2 TAGCCGCAGTCGCGCCTCGAGCTAC 409

**** * * * *

Alignment #6

Hu_400+ TTCTGACACAACCTGCTACACATCTTGGATCCC--ACTTGTCAAGCCTGGCCCTAGGCCT 58

Mm_409+S3 -------TAGTCACGCGTAGCTTATTGGGTAACCTGCCTACACAGTACGAGCGTAGTAAA 53

* ** * * **** * * * * ** * * ***

Hu_400+ CTCAGGGGTGGGTACATCAAGTGAGGAAGTCACACTGTCAGGGCAGAA----AGAG-GGG 113

Mm_409+S3 AATGCGGGGGGCTGA-ATACGTACCTCATTGCACCTCCGAGGCGCATGCGCGAGCGTCCC 112

*** ** * * ** * * ** *** ** *

Hu_400+ CTGGGTGGGGCCGGACAGGCTCTGCTGGCTCAGGATCCCGGCAGGACAGGCTGCCC**AACC** 173

Mm_409+S3 CCGGGGGGTGCCGAACGGCATGCCATGCACCAT------TCACGTGGACACTTCCA**AACC** 166

* *** ** **** ** * * ** ** * * ** ** ********

Hu_400+ **C**AGCCCCAAAACTCCCATCTTCTGTCCAACGCCCAAGC----CTGCTTCCTCCCTGCCCC 229

Mm_409+S3 **C**CG-GACTGGTATCCAATCACCGCTAGATAGCTCGGGGCGGCACGGATTCGATCTTCGCC 225

***** * * *** *** * * * ** * * * * * ** * **

Hu_400+ ACCTCACCTCACACTGCCCAGACTGGTGGGGCTGCATTTTTGGGGCCGGTGCCA**GGTGTG** 289

Mm_409+S3 GTGTCGCAAGGCCG----------AGTCGATGCTCGGGTATCACACCTAAGACT**GGTGTG** 275

** * * ** * * * * ** * * **********

Hu_400+ ATGGAGGACAGCCCAGGTCAG-------------GAGGTCAGGGCCCCGTTTCCTCCC-A 335

Mm_409+S3 CAGTAATACAACGGTCGTGCGCAGACCCTTTAAACGGTTCGCTGCCACGCTTCGGTTCGG 335

* * *** * ** * * ** *** ** *** *

Hu_400+ AGCCAGGATG----ACTCAGTGATGGACAGACTAGGCCACAAGGGGC--CGAACACAAAG 389

Mm_409+S3 GTACAGAATCGAGCTCTCTTCTCTCGCCAGCCGCAAATATCACCCGCGTCACACGTTTTA 395

*** ** *** * * *** * * * ** * **

Hu_400+ CCAAATTAGGG--- 400

Mm_409+S3 CCAGATTGACGCAC 409

*** *** *

**Alignment #7.** Shown below is the pair-wise alignment of the core human *ppl* enhancer (Hu_400+) aligned to the 467 bp enhancer block from zebrafish (Zf_467+). The 467 bp zebrafish sequence corresponds to the enhancer fragment with the highest similarity to the core of the human enhancer. These sequences correspond to the plus strand (+) sequences located upstream of *ppl*, which is transcribed to the left in each genome.

Hu_400+ TTCTGACACAACCTGCTACACATCTTGGAT-------C**CCACT**-TGTCAAGCCTGGCCCT 52

Zf_467+ ATCTCATTCAAAGAATGTCAAGCACTGTAGTCACCGTG**CCACT**AAATGAACCATGGCACT 60

*** * *** ** ** * ********* * ** * **** **

Hu_400+ AG-GCCTCTCAGGGGTGGGTACATCAAGTGAGGAAGTCACACTGTCAGGGCAGAAAGAG- 110

Zf_467+ AAACCCTATGAAGTGATTGTTTTCTTTGTCAGGCAGTATCACTTACTCTAAAACCTGAGT 120

* *** * * * * ** ** *** *** **** * * ***

Hu_400+ -GGGCTGGGTGGGGC------------CGGACA-GGC**TCTGC**TGGCTCAGGATCCCGGCA 156

Zf_467+ GAGGCACATTTGCAACATTCTTATTAAATGCCAGTCT**TCTGC**GACCTGTGACACACTTCA 180

*** * * * ** ********* ** * * * **

Hu_400+ GGACAGGCTGCCCAACCC----AGCCCCAAAACTCCCATCTTCTGTCCAACGCCCAAGCC 212

Zf_467+ ATACTGAGTGTGTAAGATGGGATGGTGTAGGCCTACTAGCTGATGTCTCAC---TAAATC 237

** * ** ** * * ** * * ** **** ** ** *

Hu_400+ TGCTTCCTCCCTGCCCCACCTCACCTCACACTGCCCAGA-----CTG**GTGGG**GCTGCATT 267

Zf_467+ TGTCAGATTAGTGCAGAACTGTTCCGTTCCATGCCTGGAAGTTTGTA**GTGGG**CCTGAAGA 297

** * *** ** ** * **** ** * ********* *** *

Hu_400+ TT--TGGGGCCGGTGC**CAGGT**GT----GATGGAGGACAGCCCAGGTCAGGAGG------- 314

Zf_467+ CCTTGATTTGCTTTGT**CAGGT**ATGTTTAATTAAGGTTAGCGCTAAACTCTACATGACAAT 357

* ** ********* * ** *** *** * * *

Hu_400+ ---------------TCAGGGCCCCGTTTCCTCCCAAGCCAGGATGACTCAGTGATGGAC 359

Zf_467+ GGTCCTCCAGGAACAGAAAGTTCACCTTCAATAGACAGCAATGCATGTTTACTTTTCTGC 417

* * * * ** * *** * * * * * * *

Hu_400+ AGACTAGGC--------CACA**AGGGG**CCGAACACAAAGCCA-AATTAGGG 400

Zf_467+ AAAGTACCAAACCTCACCGCT**AGGGG**GAGTCGAATAGTTTTGACTGAGGC 467

* * ** * * ********* * * * * * ***

**Alignment #8.** Shown below is the pair-wise alignment of the core human *ppl* enhancer (Hu_400+) aligned to the reverse complement of the 467 bp block from zebrafish (Zf_409-) used in Alignment #7.

Hu_400+ --TTCTGAC----ACAACCTGC------TACACATCTTGGATCCCACTTGTCAAGCCTGG 48

Zf_467- GCCTCAGTCAAAACTATTCGACTCCCCCTAGCGGTGAGGTTTGGTACTTTGCAGAAAAGT 60

** * * * * * ** * * * **** ** *

Hu_400+ CCCTAGGCCT----CTCA--GGGGTGGGTACATC-AAGTGAGGAAGTCACAC**TGTCA**GGG 101

Zf_467- AAACATGCATTGCTGTCTATTGAAGGTGAACTTTCTGTTCCTGGAGGACCAT**TGTCA**TGT 120

* ** * ** * * * ** * * * ** ** ********* *

Hu_400+ CAG------------------AAAGAGGGGCTGGGTGGGGCCGGACAGGCTCTGCTGGCT 143

Zf_467- AGAGTTTAGCGCTAACCTTAATTAAACATACCTGACAAAGCAAATCAAGGTCTTCAGGCC 180

* * * * ** ** * *** * ***

Hu_400+ CAGGATCC-CGGCAGGACAGGCTGCCCAACCCA--GCCCCAAAACTCCCATCTTCTGTCC 200

Zf_467- CACTACAAACTTCCAGGCATGGAACGGAACAGTTCTGCACTAATCTGACAGATTTAGTGA 240

** * * * * ** * * *** * * ** ** ** ** **

Hu_400+ AACGCCCAAGCCTGCTTCCTCCCTGCCCCACCTCACCTCACACTGCCCAGACTGGTGGGG 260

Zf_467- GACATCAGCTAG-----TAGGCCTACACCATCCCATCTTACACACTCAGTATTGAAGTGT 295

** * *** * *** * ** ** **** * * ** * *

Hu_400+ CTGCATTTT--------TGGGGCCGGTGCCAGGTGTGATGGAGGACAGCCCAGG**TCAGG**A 312

Zf_467- GTCACAGGTCGCAGAAGACTGGCATTTAATAAGAATGTTGCAAATGTGCCTCAC**TCAGG**T 355

* * *** * * * ** ** * *** *********

Hu_400+ GGTCAGGGCCCCGTTTCCTCCCAAGCCAGGATGACTCAGTGATGGACAGACTAGGCCACA 372

Zf_467- TTT--------------AGAGTAAGTGATACTGCCTGACAAAGAAAACAATCACTTCATA 401

* *** * ** ** * * * * * ** *

Hu_400+ AGGGGCCGAACACAAAGCCAAATTAGGG-------------------------------- 400

Zf_467- GG-GTTTAGTGCCATGGTTCATTTAGTGGCACGGTGACTACAGTGCTTGACATTCTTTGA 460

* * ** * * **** *

Hu_400+ ------- 400

Zf_467- ATGAGAT 467

**Alignment #9.** Shown below is the pair-wise alignment of the core human *ppl* enhancer (Hu_400+) aligned to the reverse sequence of the 467 bp enhancer block from zebrafish (Zf_467R). This alignment constitutes a negative control as the reverse sequence is a non-biological sequence.

Hu_400+ ---------TTCTGA-----------------------CACAACCTGCTACACATCT--- 25

Zf_467R CGGAGTCAGTTTTGATAAGCTGAGGGGGATCGCCACTCCAAACCATGAAACGTCTTTTCA 60

** *** ** * * ** ** * *

**7-mer**

Hu_400+ ------------------TGGATC**CCACTTG**TCAAGCCTGGCCCTAGGCCTCTCAGGGGT 67

Zf_467R TTTGTACGTAACGACAGATAACTT**CCACTTG**AAAGACAAGGACCTCCTGGTAACAGTACA 120

* * *********** * * ** *** * ***

Hu_400+ GGGTACATCAAGTGAGGAAGTC---------------ACACTGTCAGGG**CAGAA**AGAGGG 112

Zf_467R TCTCAAATCGCGATTGGAATTAATTTGTATGGACTGTTTCGTTTAGTTC**CAGAA**GTCCGG 180

* *** * **** * * * ********* **

Hu_400+ GCTGGGTGGGGCCGGACAGGCTCTGCTGGCTCAGGATCCCGGCAGGACAGGCTGCCCAAC 172

Zf_467R GTGATGTTTGAAGGTCCGTACCTTGCCTTGTCAAGAC-----GTGATTAGACTGTCTAAA 235

* ** * * * * *** *** ** * ** *** * **

Hu_400+ CCAGCCCCAAAACTCCCATCTTCTGTCCAACGCCCAAGCCTGCTTCCTCCCTGCC--CCA 230

Zf_467R TCACTCTGT----------AGTCGATCATCCGGATGTGGTAGGGTAGAATGTGTGAGTCA 285

** * ** ** ** * * * ** **

Hu_400+ CCTCACCTCACACTGCCCAGACTG--GTGGGGCTGCATTTTT---GGGGCCGGTGCCAGG 285

Zf_467R TAACTTCACACAGTGTCCAGCGTCTTCTGACCGTAAATTATTCTTACAACGTTTACACGG 345

* * **** ** **** * ** * *** ** * * * **

Hu_400+ TGTGATGGAGGACAGCCCAGGTCAGGAGGTCAGGGCCCCGTTTCC-TCCCAAGCCAGGAT 344

Zf_467R AGTGAGTCCAAAA--TCTCATTCACTA--TGACGGACTGTTTCTTTTGTTAGTGAAGTAT 401

**** * * *** * * * ** * ** * * ** **

Hu_400+ GACTCAGTGATGGACAGACTAGGCCACAAGGG---------GC**CGAAC**ACAAAGCCAAAT 395

Zf_467R CCCAAATCACGGTACCAAGTAAATCACCGTGCCACTGATGTCA**CGAAC**TGTAAG-AAACT 460

* * * ** * ** *** * ********* *** ** *

Hu_400+ TAGGG-- 400

Zf_467R TACTCTA 467

**

**Alignments #’s 10, 11, and 12.** Shown below are three pair-wise alignments of the core human *ppl* enhancer (Hu_400+) aligned to one of three (S1, S2, and S3) different Fisher-Yates shuffled sequences of the 467 bp plus-strand block from zebrafish (Zf_467+S1/S2/S3). These pair-wise alignments serve as negative controls.

Alignment #10

Hu_400+ ---------------------------------TTCTGACACAACCTGCTACACATCTTG 27

Zf_467+S1 AACCGTGTGGCATTCTTCGGTGAAACCGGTGATTTGTACCTCACTGAATCAAGCATTATA 60

** * * ** * *** *

Hu_400+ GATCCCACTTGTCAAGCCTGGCC------CTAGGCCTCTCAGGGGTGGGTACAT------ 75

Zf_467+S1 TAGGCAAACCCCTACGGCTAGCCCAATCAATGCGTATATCTTGAGTCTGTATTGTAATTA 120

* * * * * ** *** * * * ** * ** ***

Hu_400+ -CAAGTGA**GGAAGT**CACA--CT**GTCAG**GGCAGAAAGAGGGGCTGGGTGGGGCCGGACAGG 132

Zf_467+S1 ATCCTTGG**GGAAGT**AATCCCCG**GTCAG**CTCACAAAGC--TACGGGGTCTGTAATAAGTCA 178

** ********** * * ********* ** **** * **** * *

Hu_400+ CTCTGCTGGCT-----------CAGGATCC----CGGCAGGACAGGCTGCCCAACCCAGC 177

Zf_467+S1 CTACGTTGCTTAGAAGCAGCTTTAGCATAGATGTAGTCTCTACAGTGTTAAAGCTCCACT 238

** * ** * ** ** * * **** * ***

Hu_400+ C------------CCAAAACTCCCATCTTCTGTCCAACGCCCAAGCCTGCTTCCTCCCT- 224

Zf_467+S1 CACTAGAATGATCTCAGATTATAATTCTTGTGTGTAACGTTAGCCCATCGTGTTGACCAC 298

* ** * **** *** **** * * * **

Hu_400+ --GCCCCACCTCACCTCACACTGCCCAGACTGGTGGGGCTGCATTTTTGGGGCCGGTG-- 280

Zf_467+S1 GCTGCACTATCCAGTCCAGACGTTTGAAATTGCTGTGCATAAGTTATATCACTCCGATAG 358

* * ** ** ** * * ** ** * * ** * * *

Hu_400+ CC**AGGTG**TGA-TGGAGGACAGCCCAGGTCAG**GAGGT**CAGGGCCCCGT**TTCCT**CCCAAGCC 339

Zf_467+S1 GG**AGGTG**GGTCTGTCTAACACTGAAGTTCTT**GAGGT**TAAA------C**TTCCT**G---AATC 409

********* * ** *** ** ** ********* * ********* * *

Hu_400+ AGGATGACTCAGTGATGGACAGACTAGGCCACAAGGGGCCGAACACAAAGCCAAATTAGG 399

Zf_467+S1 AAATGGAATAAATACGTACACTTTTAGTACTTCGAAGTGC---TGCAACGCAGTGTTGCA 466

* ** * * * *** * * * *** ** **

Hu_400+ G 400

Zf_467+S1 A 467

Alignment #11

Hu_400+ -TTCTGACAC**AACCT**--GCTACAC---------------ATC**TTGGAT**CCCACTTGTCAA 42

Zf_467+S2 CAAAGGAGAG**AACCT**TAGGAACACTTATAGCACGGGAATTTT**TTGGAT**TCCGTTAATGGA 60

** * ********* * **** * ********** ** * * *

Hu_400+ G-CCTGGCCCTAGGCCTCTCAGGGGTGGGTAC----ATCAAGT----------GAGGAAG 87

Zf_467+S2 ACTGTGCCTATACTAATATCAAACTTGATGTGATATTTAACCTTTAAAATACAGTGAAAG 120

** * ** * *** ** * * * * * ***

Hu_400+ TCACACTGTCAGGGCAGAAAGAG----GGGCTGGGTGGGGCCGGACAGGCTCTGCTGGCT 143

Zf_467+S2 ATGTACTTATTATTTATATAGATTGTATGTCTCATTAACGCAAACCGTTGTCTACTTCCC 180

*** * * *** * ** * ** * *** ** *

Hu_400+ CAGGATCCCGGCAGGACAGGCTGCCCAACCCAGCCCCAAAACTCCCATCTTCTGTCCAAC 203

Zf_467+S2 -----TATTGGTTGACTAAGCTACACGTCTTTGCCCGT-----ACACTGTTAGGTATACA 230

* ** * * *** * * * **** * * ** ** *

Hu_400+ GCCCAAGCCTGCTTCCTCCCTGCCCCACCTCACCTCACACTGCCCAGACTGGTGGGGCTG 263

Zf_467+S2 AACCA---------------------ATTAGGTCTCCCGCTCCAGAGTAAGTGGGGGTTG 269

*** * *** * ** * ** * **** **

Hu_400+ CATTTTTGGGGCCGGTGCCAGGTGTGATGGAGGACAGCCCAGGTCAGGAGGTCAGGGCCC 323

Zf_467+S2 AAATCATCGGAGCGACGCGTCATATAGTTTCTTAC---GGAGATAGGGCGCAAAGTGCAA 326

* * * ** ** ** * * * ** ** * ** * ** **

Hu_400+ CGTT---TCCT-----CCCAAGCCAG-------GATGACTCAGTGATGGACAGACTAGGC 368

Zf_467+S2 CTTCGATTAATATGCCGCCCATAGAACATTCAACATTACTTCGAAGCTGCCCCACCCCAC 386

* * * * ** * * ** *** * * * ** *

Hu_400+ CACAA---------GGGGCCGA---ACACAAAGCCAAA**TTAGG**G---------------- 400

Zf_467+S2 GTGCAGGGTCTTTTGGCGGCGAGAAGGTCAACTACGTT**TTAGG**TAAGATTTTCTGTGACT 446

* ** * *** *** * *********

Hu_400+ --------------------- 400

Zf_467+S2 GTTGGTCACGCCCCGAGTCCT 467

Alignment #12

Hu_400+ ---TTCTGACACAACCTGCTACACATCTTGGATCCCACTTGT----------CAAGCCTG 47

Zf_467+S3 AGGAGTAGGCAACTAAAGATGCGTATTTTAGGTTCCTCTTCAGCCCAAACTACACACCCT 60

* ** * * * ** ** * * ** *** ** **

Hu_400+ GCCCTAGGCCTC**TCAGGG**GTGGGTACATCAAGTGAGGAAGTCACACTGTCAGGGCAGAAA 107

Zf_467+S3 AACGTGGGCT--**TCAGGG**ATGTCTTTTGCATCTTACCTAATTTACACGGAGCCGCAGTAA 118

* * *** ********** ** * ** * * * * * **** **

Hu_400+ GAGGGGCTGGGTGGGGCCGGACAGGCTCTGCTGGCTCAGGAT-CCCGGCAG-GACAGGCT 165

Zf_467+S3 TTAAGTATACTTTTGCAGAAGTAGTCTGCCCATAAATTGAAAGCTGGTCGCAGCTTGTTA 178

* * * * ** ** * * * * * * * *

**7-mer**

Hu_400+ **GCCCAAC**CCAGCCCCAAAACTCC**CATCTT**CTGTCCAA--CGCCCAAGCCTGCTTCCTCCC 223

Zf_467+S3 **GCCCAAC**TCGTATCCAGTCATTG**CATCTT**GTGACTTTGCAGGCTAAGCTCCCCTCATAAG 238

*********** * *** * ********** ** * * * **** * ** *

Hu_400+ TG----CCCCACCTCACCTCACACTGCCCAGACTGGTG**GGGCT**GCATTTTT-GGG-GCCG 277

Zf_467+S3 GGGTTTACTCGCTCATCCTATGCGTAATACTCATACAT**GGGCT**TCAAGTTTAGGTAGCCC 298

* * * * *** * * ********* ** *** ** ***

Hu_400+ GTGCCAGGTGTG-------ATGGAGGACAGCCCAGGTCAGGAGGTCAGGGCCCCGTTTCC 330

Zf_467+S3 ATTGACGGTAGAATTACCAATTGAATATTGAGGATTAAATATGGCACGGGGACCTAGACG 358

* *** ** ** * * * * ** *** ** *

Hu_400+ TCCCAA-GCCAGGATGACTCAGTGATGGACAGACTAGGCCACAAGGGGCCGAACACAAAG 389

Zf_467+S3 TCACAAAGTCTTCGTTAATCT-AGGTGTACAATCTTTGTACTCATGTGGCGTCCGAAAGG 417

** *** * * * * ** * ** *** ** * * * * ** * ** *

Hu_400+ CCAAATTAGGG--------------------------------------- 400

Zf_467+S3 ATAAAATGAGGAAATGTTGGTCTTCAACAAACACTTCGTTCATAAGGTTT 467

*** * **

**Alignments # 13.** For reference, shown below is a pair-wise alignment of the core human *ppl* enhancer (Hu_400+) aligned to the full-length 812 bp enhancer from the zebrafish *ppl* locus (Zf_812+).

Hu_400+ ------------------------------------------------------------ 0

Zf_812+ CCCACCTACATTCTGGAACGGGGAGGCTCGGAGAAAAACCAGCCAATCCTGCCTGGCCCC 60

Hu_400+ ------------TTCTGACACAACCTGCTACACATCTTGGATC----------------C 32

Zf_812+ AGACATTTATTATCATGAAAAAGAGTGTTAAACATGCTTTTAAAGAAGTCATTGTGTTGT 120

* *** * * ** ** **** *

Hu_400+ CACTTGT--------------------------------------CAAGCCTGGCCCTAG 54

Zf_812+ GACTAATAAGATGTGTTGTTAAAAGTGTCTCTTTAGGGAATCTGCCATCAAACGCTGTAA 180

*** * ** ** **

Hu_400+ GCCTCTCAGGGGTGGGTACATCAAGTGAGGAAGTCACACTGTCAGGGCAGAAAGAGGGGC 114

Zf_812+ TTCTCCTCGGCTTG-----CTTAAGACAGAAAGGAACATTGCCTTACAGATAAG-GAGGA 234

*** ** ** * *** ** *** *** ** * *** * **

Hu_400+ TGGGTGGGGCCGGACAGGCTCTGCTGGCTCA----GGATCCCG-GCAGGACAGGCTGCCC 169

Zf_812+ ATGATGACAGAGCAACAGTAATGCACACTGATCTACTATACTATCTGAAACATGCAGCCA 294

* ** * * * *** ** * ** * *** ** ***

Hu_400+ AACCCAGCCCCA-----AAACTCCCATCTTCTGTCCAACGCCCAAGC------------- 211

Zf_812+ CACCCATTCAGTCACTCTTTCTGTCATTTTCCACCTCACACCGTGATCTCATTCAAAGAA 354

***** * ** *** *** * ** **

Hu_400+ ---------CTGCTTCCTCCCTGCCCCACC---TCACCTCACACTGCCCAGACTGGTGGG 259

Zf_812+ TGTCAAGCACTGTAGTCACCGTGCCACTAAATGAACCATGGCACTAAACCCTATGAAGTG 414

*** * ** **** * * * **** * ** * *

Hu_400+ GCTGCATTTTTGGG--GCCGGTGCCAGGTGTGATGGAGGACAGCCCAGGTCAGGAGGTCA 317

Zf_812+ ATTGTTTTCTTTGTCAGGCAGTATCACTTA-----CTCTAAAACCTGAGTGAGGCACATT 469

** ** ** * * * ** ** * * * ** ** ***

Hu_400+ GGGCCCCGTTTCCTCCCAAGCCAGGATGACTCAGTGATGGACAGACTAG----------- 366

Zf_812+ TGCAACATTCTTATTAAATGCCAGTCTTCTGCGACCTGTGACACACTTCAATACTGAGTG 529

* * * * * * ***** * * **** ***

Hu_400+ -G---------------------------------------------------------- 367

Zf_812+ TGTAAGATGGGATGGTGTAGGCCTACTAGCTGATGTCTCACTAAATCTGTCAGATTAGTG 589

*

Hu_400+ ------------------------------------------------------------ 367

Zf_812+ CAGAACTGTTCCGTTCCATGCCTGGAAGTTTGTAGTGGGCCTGAAGACCTTGATTTGCTT 649

Hu_400+ ----------------------------------------CCACAAGGGG---------- 377

Zf_812+ TGTCAGGTATGTTTAATTAAGGTTAGCGCTAAACTCTACATGACAATGGTCCTCCAGGAA 709

**** **

Hu_400+ CCGAACACAAAGCCAAATTAGGG------------------------------------- 400

Zf_812+ CAGAAAGTTCACCTTCAATAGACAGCAATGCATGTTTACTTTTCTGCAAAGTACCAAACC 769

* *** * * * ***

Hu_400+ ------------------------------------------- 400

Zf_812+ TCACCGCTAGGGGGAGTCGAATAGTTTTGACTGAGGCTTGGAA 812

**Alignments # 14.** For reference, shown below is a pair-wise alignment of the full-length human *ppl* enhancer (Hu_489+) aligned to the full-length 812 bp enhancer from the zebrafish *ppl* locus (Zf_812+). Relative to the 400 bp core enhancer used in the majority of pair-wise alignments, the full-length enhancer sequences overlaps a non-conserved Alu element (AluJr4 sub-family) on the *ppl*-proximal side and a MIR-element on the *ppl*-distal side.

Hu_489+ ------------------------------------------------------------ 0

Zf_812+ CCCACCTACATTCTGGAACGGGGAGGCTCGGAGAAAAACCAGCCAATCCTGCCTGGCCCC 60

Hu_489+ ------------TTCTGACACAACCTGCTACACATCTTGGATC----------------C 32

Zf_812+ AGACATTTATTATCATGAAAAAGAGTGTTAAACATGCTTTTAAAGAAGTCATTGTGTTGT 120

* *** * * ** ** **** *

Hu_489+ CACTTGT--------------------------------------CAAGCCTGGCCCTAG 54

Zf_812+ GACTAATAAGATGTGTTGTTAAAAGTGTCTCTTTAGGGAATCTGCCATCAAACGCTGTAA 180

*** * ** ** **

Hu_489+ GCCTCTCAGGGGTGGGTACATCAAGTGAGGAAGTCACACTGTCAGGGCAGAAAGAGGGGC 114

Zf_812+ TTCTCCTCGGCTTG-----CTTAAGACAGAAAGGAACATTGCCTTACAGATAAG-GAGGA 234

*** ** ** * *** ** *** *** ** * *** * **

Hu_489+ TGGGTGGGGCCGGACAGGCTCTGCTGGCTCA----GGATCCCG-GCAGGACAGGCTGCCC 169

Zf_812+ ATGATGACAGAGCAACAGTAATGCACACTGATCTACTATACTATCTGAAACATGCAGCCA 294

* ** * * * *** ** * ** * *** ** ***

Hu_489+ AACCCAGCCCCA-----AAACTCCCATCTTCTGTCCAACGCCCAAGC------------- 211

Zf_812+ CACCCATTCAGTCACTCTTTCTGTCATTTTCCACCTCACACCGTGATCTCATTCAAAGAA 354

***** * ** *** *** * ** **

Hu_489+ ---------CTGCTTCCTCCCTGCCCCACC---TCACCTCACACTGCCCAGACTGGTGGG 259

Zf_812+ TGTCAAGCACTGTAGTCACCGTGCCACTAAATGAACCATGGCACTAAACCCTATGAAGTG 414

*** * ** **** * * * **** * ** * *

Hu_489+ GCTGCATTTTTGGG--GCCGGTGCCAGGTGTGATGGAGGACAGCCCAGGTCAGGAGGTCA 317

Zf_812+ ATTGTTTTCTTTGTCAGGCAGTATCACTTA-----CTCTAAAACCTGAGTGAGGCACATT 469

** ** ** * * * ** ** * * * ** ** ***

Hu_489+ GGGCCCCGTTTCCTCCCAAGCCAGGATGACTCAGTGATGGACAGACTAG----------- 366

Zf_812+ TGCAACATTCTTATTAAATGCCAGTCTTCTGCGACCTGTGACACACTTCAATACTGAGTG 529

* * * * * * ***** * * **** ***

Hu_489+ -G---------------------------------------------------------- 367

Zf_812+ TGTAAGATGGGATGGTGTAGGCCTACTAGCTGATGTCTCACTAAATCTGTCAGATTAGTG 589

*

Hu_489+ ------------------------------------------------------------ 367

Zf_812+ CAGAACTGTTCCGTTCCATGCCTGGAAGTTTGTAGTGGGCCTGAAGACCTTGATTTGCTT 649

Hu_489+ ----------------------------------------CCACAAGGGG---------- 377

Zf_812+ TGTCAGGTATGTTTAATTAAGGTTAGCGCTAAACTCTACATGACAATGGTCCTCCAGGAA 709

**** **

Hu_489+ CCGAACACAAAGCCAAATTAGGG------------------------------------- 400

Zf_812+ CAGAAAGTTCACCTTCAATAGACAGCAATGCATGTTTACTTTTCTGCAAAGTACCAAACC 769

* *** * * * ***

Hu_489+ ------------------------------------------- 400

Zf_812+ TCACCGCTAGGGGGAGTCGAATAGTTTTGACTGAGGCTTGGAA 812

**Alignments # 15.** For reference, shown below is a three-way multiple sequence alignment of the core human *ppl* enhancer (Hu_400+), the homologous 409 bp block from mouse *ppl* locus (Mm_409+), and the 467 bp block from the zebrafish *ppl* locus (Zf_467+), which is located in a similar position as the mammalian enhancer. Highlighted are two of the 18 conserved mammalian blocks with some similarity to zebrafish sequences.

Hu_400+ --TTCTGACACAACCTGCTACACAT------CTTGGATCCCACTTGTCAAGCCTGGCCCT 52

Mm_409+ --CTATCCCACAGACTGGTGACCCT-GGAG-CTCAGACACTCCACTGGAGAGCTATCCCT 56

Zf_467+ ATCTCATTCAAAGAATGTCAAGCACTGTAGTCACCGTGCCACTAAATGAACCATGGCACT 60

* ** * ** * * * * * * * **

**b. 10-mer**

Hu_400+ AGGCCTCTCAGG------GGTGGGTACATCAAGTG**AGGAAGTCAC**ACTGTC----AGGGC 102

Mm_409+ GGGCCTCTCAAATTCACCATGAGAGCTTCTCTGCA**AGGAAGTCAC**CCCATC----AGGGC 112

Zf_467+ AAACCCTATGAAG-----TGATTGTTTTCTTTGTC**AGG**C**AGT**AT**C**ACTTACTCTAAAACC 115

** * ***** *** *** * * * *

**e. 9-mer f.**>>

Hu_400+ AGAAAGAGGGGCT------GGGTG----GGGCCGG**ACAGGCTCT**GC-TGGCTCAGG**ATCC** 151

Mm_409+ AGA--GTGGTTCT------AGGTG----GGGCTAA**ACAGGCTCT**ACCAGGCACAGT**ATCC** 160

Zf_467+ TGAGTGAGGCACATTTGCAACATTCTTATTAAATGC**CAG**T**CTT**CTGCGACCTGTGACA**C**A 175

** * ** * * ******* ****** * * *****

> **g.**>>> **h. 7-mer**

Hu_400+ **C**G**GCAGG**A**CAG**G**CTGCCCA**ACCCA----GCCCCAAAACTCCCATCTTCTGTCCAACGCCC 207

Mm_409+ **C**T**GCAGG**G**CAG**A**CTGCCCA**GCCTC----CTGTTCACG--------------CCCATGCTT 202

Zf_467+ **C**TT**CA**ATA**C**T**G**AG**TG**TGT**A**AGATGGGATGGTGTAGGCCTACTAGCTGATGTCTCACTAAA 235

***** ****** ***** ***** ****** ***** * *

Hu_400+ -AAGCCTGCTTCCTCCCTGCCCC---------------ACCTCACCTCACACTGCCCAGA 251

Mm_409+ -TTTCCTGCCTGCTGCCTGCTCCTCCCCTTCTGGGCCGTGCCAGGCTCATGCAGAGCAGA 261

Zf_467+ TCTGTCAGATTAGTGCAGAACTGTTCCGTTCCATGCCTGGAAGTTTGTAGTGGGCCTGAA 295

* * * * * * * *

**l.**>>> **m.**>>>

Hu_400+ CTGGTGGGGCT**GCATT**T**TTGGG**GC---------------CGGTGCCAGGTGTGATGGA-- 294

Mm_409+ CCAGTACAGCC**GCATT**C**TTGGG**CAA--------------GTGTCTCTGGTGTGAATGACC 307

Zf_467+ GACCTTGATTT**GC**T**TT**G**T**CA**GG**TATGTTTAATTAAGGTTAGCGCTAAACTCTACATGACA 355

* ****** ****** ***** ****** * * **

Hu_400+ ---GGACAGCCCAGGTCAGGAGGTCAGGGCCCCGTTTCCTCCCAAGCCAGGATGACTCAG 351

Mm_409+ CTGGGGCTGGTCAGGTCAGAAGGTCAGGGCCTTGTTCCCTCCAGTGCCCAAGTGATA--- 364

Zf_467+ ATGGTCC------TCCAGGAACAGAAAGTTCACCTTCAATAGACAGCAATGCATGTTTAC 409

* * * * * * * ** * **

Hu_400+ TGATGGACAGACTAGGCCA-------CAAGGGG-CCGAACACAAAGCCAAATTAGGG- 400

Mm_409+ -----GATGAGTCAGGCTA-------CAAAGGGGCAGAACAAGAAACCAAACCGAGT- 409

Zf_467+ TTTTCTGCAAAGTACCAAACCTCACCGCTAGGGGGAGTCGAATAGTTTTGACTGAGGC 467

* * *** * * * * *

* 96 count (3-way identical characters)

- 158 count (null characters)

**Alignments # 16.** For reference, shown below is a three-way multiple sequence alignment of the core human *ppl* enhancer (Hu_400+), the homologous 409 bp block from mouse *ppl* locus (Mm_409+), and the reverse complement of the 467 bp block from the zebrafish *ppl* locus (Zf_467-). This alignment serves as a control alignment for Alignment #15, which features far fewer insertions of null characters (158 dashes versus 227 dashes). However, the number of alignment columns with 3-way identity is similar in both (96 and 97 * columns).

Hu_400+ ------TTCTGACACAACCTGCTACACAT----CTTGGATC--CCACTTGTCAAGCCTGG 48

Mm_409+ ------CTATCCCACAGACTGGTGACCCTGGAGCTCAGACA--CTCCACTGGAGAGCTAT 52

Zf_467- GCCTCAGTCAAAACTATTCGACTCCCCCTAGCGGTGAGGTTTGGTACTTTGCAGAAAAGT 60

* * * * * * * * * *

**a. 9-mer** **c. 10-mer**

Hu_400+ CCCTA**GGCCTCTCA**GG------GGTGGGTACATCAAGTG-AGGAAGTCACACTG**TCAGGG** 101

Mm_409+ CCCTG**GGCCTCTCA**AATTCACCATGAGAGCTTCTCTGCA-AGGAAGTCACCCCA**TCAGGG** 111

Zf_467- AAACAT**GC**A**T**TG**C**TGTCTATTGAAGGTGAACTTTCTGTTCCTGGAGGACCATTG**TCA**T-**G** 119

****** ***** ***** * * ** * ******* *****

**e. 9-mer**

Hu_400+ **CAGA**AAGAGGGGCT-------------------GGGTGGGGCCGG**ACAGGCTCT**G**C**-TGG 141

Mm_409+ **CAGA**--GTGGTTCT-------------------AGGTGGGGCTAA**ACAGGCTCT**A**C**CAGG 150

Zf_467- T**AGA**GTTTAGCGCTAACCTTAATTAAACATACCTGACAAAGCAAAT**CA**A**G**G**TCT**T**C**AGGC 179

******* * ** * ** ****** ***** ******* ***** *

Hu_400+ CTCAGGATCCCGGCAGGACAGGCTGCCCAACC--CAGCCCCAAAACTCCCA--TCTTCTG 197

Mm_409+ CACAGTATCCCTGCAGGGCAGACTGCCCAGCC--TCCTGTTCACG--------------- 193

Zf_467- CCACTACAAACTTCCAGGCATGGAACGGAACAGTTCTGCACTAATCTGACAGATTTAGTG 239

* * * * ** * * * *

Hu_400+ TCCAACGCCCAAGCCTGCTTCCTCCCTGCCCC---------------ACCTCACCTCACA 242

Mm_409+ -CCCATGCTTTTTCCTGCCTGCTGCCTGCTCCTCCCCTTCTGGGCCGTGCCAGGCTCATG 252

Zf_467- AGACATCAGCTAGTAGGCC-------TACACCATCCCATCTTACACACTCAGTATTGAAG 292

* ** * * ** * * *

**n. 7-mer**

Hu_400+ C---TGCCCAGACTGGTGGGGCTGCATTTTTGGGGC-CGGTGC**CAGGTGTGA**TG**GA**----- 294

Mm_409+ C---AGAGCAGACCAGTACAGCCGCATTCTTGGGCAAGTGTCT**C**T**GGTGTGA**A**TGA**----- 305

Zf_467- TGTGTCACAGGTCGCAGAAGACTGGCATTTAATAAGAATGTTG**CA**AA**TGTG**CC**T**C**A**CTCAG 353

* * * * * * ** ***** ******** *****

**p. 11-mer**

Hu_400+ ---------------GGACAGCCCAGGTCAGG-----------------**AGGTCAGGGCC** 322

Mm_409+ ----------CCCTGGGGCTGGTCAGGTCAGA-----------------**AGGTCAGGGCC** 338

Zf_467- GTTTTAGAGTAAGTGATACTGCCTGACAAAGAAAACAATCACTTCATAGG**G**T**T**T**AG**T**GCC** 413

* * ** ***** ***** ****** *******

Hu_400+ CCGTTTCCTCCCAAGCCAGGATG-ACTCAGTGATGGACAGACTAGGCCACAAGGGG-CCG 379

Mm_409+ TTGTTCCCTCCAGTGCCCAAGTG-ATA--------GATGAGTCAGGCTACAAAGGGGCAG 388

Zf_467- ATGGTTCATTTAGTGGCACGGTGACTACAGTGCTTGACATTCTTTGAATGA--------- 464

* * * * * * ** ** * *

Hu_400+ AACACAAAGCCAAATTAGGG 400

Mm_409+ AACAAGAAACCAAACCGAGT 409

Zf_467- ----------------GAT- 467

* 97 count (3-way identical characters)

- 227 count (null characters)

**Alignments # 17.** For reference, shown below is a three-way multiple sequence alignment of the core human *ppl* enhancer (Hu_400+), the homologous 409 bp block from mouse *ppl* locus (Mm_409+), and the (non-biological) reverse sequence of the 467 bp block from the zebrafish *ppl* locus (Zf_467R). This alignment serves as a control alignment for Alignment’s #15 and #16, which features far fewer insertions of null characters (158 dashes versus 227 dashes). While the number of alignment columns with 3-way identity is similar (99 * columns versus 96 and 97 * columns in Alignments #15 and #16, respectively), Alignment #16 features many more insertions of null characters (248 dashes inserted versus 158 and 227 dashes in Alignments #15 and #16, respectively).

Hu_400+ -------TTCTGACACAACCTGCTACACAT------------CTTGGATCCCACTTGTCA 41

Mm_409+ -------CTATCCCACAGACTGGTGACCCTGG--------AGCTCAGACACTCCACTGGA 45

Zf_467R CGGAGTCAGTTTTGATAAGCTGAGGGGGATCGCCACTCCAAACCATGAAACGTCTTTTCA 60

* * * *** * * ** * * *

Hu_400+ AGCCTGGCCCTAGGCCTCT-----CAGG------GGTGGGTACATCAAGTGAGGAAGTCA 90

Mm_409+ GAGCTATCCCTGGGCCTCT-----CAAATTCACCATGAGAGCTTCTCTGCAAGGAAGTCA 100

Zf_467R TTTGTACGTAACGACAGATAACTTCCACTTGAAAGACAAGGACCTCCTGGTAACAGTACA 120

* * * * * * * * **

Hu_400+ CACTGTCAGGGCAGAAAGAGGGGCTGGGTGGGGCCGGACAGGCTCTGC-TGGCTCAGGAT 149

Mm_409+ CCCCATCAGGGCAGA--GTGGTTCTAGGTGGGGCTAAACAGGCTCTACCAGGCACAGTAT 158

Zf_467R T-CTCAAATCGCG----ATTGGAATTAATTTGTATGGACTGTTTCGTTTAGTTCCAGAAG 175

* * ** * * * * ** * ** * *** *

Hu_400+ CCCGGCA------GGACAGGCTGCCCAACCCAGCCCCAAAACTCCCATCTTCTGTCCAAC 203

Mm_409+ CCCTGCA------GGGCAGACTGCCCAGCCTCCTGTTCACG--------------CCCAT 198

Zf_467R TCCGGGTGATGTTTGAAGGTCCGTACCTTGCCTTGTCAAGACGTGATTAGACTGTCTAAA 235

** * * * * * * * * *

Hu_400+ GCCCAAGCCTGCTTCCTCCCTGCCCC-----------------------------ACCTC 234

Mm_409+ GCTTTTTCCTGCCTGCTGCCTGCTCCTCCCCTTCTGGGCCG--------------TGCCA 244

Zf_467R TCACT-----CTGTAGT---CGATCATCCGGATGTGGTAGGGTAGAATGTGTGAGTCATA 287

* * * * *

Hu_400+ ACCTCACA--CTGCCCAGACTG--GTGGGGCTGCATTTTTGGGGC-CGGTGCCAGGTGTG 289

Mm_409+ GGCTCATG--CAGAGCAGACCA--GTACAGCCGCATTCTTGGGCAAGTGTCTCTGGTGTG 300

Zf_467R ACTTCACACAGTGTCCAGCGTCTTCTGACCGTAAATTATTCTTACAACGTTTA------- 340

*** * *** * *** ** **

Hu_400+ ATGGA-----GGACAGCCCAGGTCAGGAGGTCAGGGCCCCGTTTCCTCCCAAGCCAGGAT 344

Mm_409+ AATGACCCTGGGGCTGGTCAGGTCAGAAGGTCAGGGCCTTGTTCCCTCCAGTGCCCAAGT 360

Zf_467R ------------------CACGGAGTGAGTCCAAAATCTCATTCACTATGACGGA---CT 379

** * ** ** * ** ** * *

Hu_400+ GACTCAGTGATGGACAGACTAGGCCACAAGG----------------------------- 375

Mm_409+ GATA--------GATGAGTCAGGCTACAAAG----------------------------- 383

Zf_467R GTTTCTTTTGTTAGTGAAGTATCCCAAATCACGGTACCAAGTAAATCACCGTGCCACTGA 439

* * * * *

Hu_400+ -GG-CCGAACACAAAGCCAAATTAGGG- 400

Mm_409+ -GGGCAGAACAAGAAACCAAACCGAGT- 409

Zf_467R TGTCACGAACTGTAAGAAACTTACTCTA 467

* **** ** *

* 99 count (3-way identical characters)

- 248 count (null characters)

**Alignments #’s 18, 19, and 20.** Shown below are three 3-way alignments of the core mammalian *ppl* enhancers (Hu_400+ and Mm_409+) aligned to one of three (S1, S2, and S3) different Fisher-Yates shuffled sequences of the 467 bp plus-strand block from zebrafish (Zf_467+S1/S2/S3). These multiple-sequence alignments serve as negative controls.

Alignment #18

Hu_400+ ----TTCTGACACAACCTGCTACACAT----CTTGGATCCC-------------ACTTGT 39

Mm_409+ ----CTATCCCACAGACTGGTGACCCTGGAGCTCAGACACT-------------CCACTG 43

Zf_467+S1 AACCGTGTGGCATTCTTCGGTGAAACCGGTGATTTGTACCTCACTGAATCAAGCATTATA 60

* * ** * * * * *

Hu_400+ CAAGCCTGGCCCTAGGCCTCTCAGG-------------------------GGTGGGTACA 74

Mm_409+ GAGAGCTATCCCTGGGCCTCTCAAATTCAC-------------------CATGAGAGCTT 84

Zf_467+S1 TAGGCAAACCCCTACGGCTAGCCCAATCAATGCGTATATCTTGAGTCTGTATTGTAATTA 120

* **** * ** *

Hu_400+ TCAAGTGA**GGAAGT**CACACTGTCAGGGCAGAAAGAGGGGCTGGGTGGGGCCGGACAGGCT 134

Mm_409+ CTCTGCAA**GGAAGT**CACCCCATCAGGGCAGA--GTGGTTCTAGGTGGGGCTAAACAGGCT 142

Zf_467+S1 ATCCTTGG**GGAAGT**AATCCCCGGTCAGCTCACAAAGCTACGGGGTCTGT--AATAAGTCA 178

********** * * ** * * * *** * ** *

Hu_400+ CTGC-TGGCTCAGGATCCCGGCAGGACAGGCTG--CCCAACCCAGCCCCAAAACTCCCAT 191

Mm_409+ CTACCAGGCACAGTATCCCTGCAGGGCAGACTG--CCCAGCCTCCTGTTCACG------- 193

Zf_467+S1 CTACGTTGCTTAGAAGCAGCTTTAGCATAGATGTAGTCTCTACAGTGTTAAAGCTCCACT 238

** * ** ** * * * ** * *

Hu_400+ C-------TTCTGTCCAACGCCCAAGCCTGCTTCCTCCCTGCCCC--------------- 229

Mm_409+ --------------CCCATGCTTTTTCCTGCCTGCTGCCTGCTCCTCCCCTTCTGGGCCG 239

Zf_467+S1 CACTAGAATGATCTCAGATTATAATTCTTGTGTGTAACGTTAGCCCATCGTG-TTGACCA 297

* * * ** * * * **

Hu_400+ ACCTCACCTCACACTGCC**CAGAC**TGGTGGGGCTGCATTTTTGGGGC-CGGT---GCCAGG 285

Mm_409+ TGCCAGGCTCATGCAGAG**CAGAC**CAGTACAGCCGCATTCTTGGGCAAGTGT---CTCTGG 296

Zf_467+S1 CG-CTGCACTATCCAGTC**CAGAC**GTTTGAA-----ATTGCTGTGCATAAGTTATATCACT 351

* * * ********* * *** ** * ** *

Hu_400+ TGTGATGGA-----GGACAGCCCAGGTCAGGAGGTCAGGGCCCCGTTTCCTCCCAAGCCA 340

Mm_409+ TGTGAATGACCCTGGGGCTGGTCAGGTCAGAAGGTCAGGGCCTTGTTCCCTCCAGTGCCC 356

Zf_467+S1 CCGATAGGGAGGTGGGTCTGTCTAACACTGAAGTTCTTGAGGTTAAA-CTTCCTGAATCA 410

* ** * * * * * ** ** * * *** *

Hu_400+ GGATGACTCA-GTGATGGACAGACTAGGCCACAAGGGG-CCGAACACAAAGCCAAATTAG 398

Mm_409+ AAGTGATA---------GATGAGTCAGGCTACAAAGGGGCAGAACAAGAAACCAAACCGA 407

Zf_467+S1 AATGGAATAAATACGTACACTTTTAGTACTTCGAAGTGCTGCAACGCAGTGTTGCAA--- 467

** * * * * * * *** *

Hu_400+ GG 400

Mm_409+ GT 409

Zf_467+S1 -- 467

* 110 count (3-way identical characters)

- 170 count (null characters)

Alignment #19

Hu_400+ ----------TTCTGACACAACCTGCTACACAT----CTT---GGAT-CCCACTTGTCAA 42

Mm_409+ ----------CTATCCCACAGACTGGTGACCCTGGAGCTC---AGAC-ACTCCACTGGAG 46

Zf_467+S2 AAAGGAGAGAACCTTAGGAACACTTATAGCACGGGAATTTTTTGGATTCCGTTAATGGAA 60

* * ** * * ** * *

Hu_400+ GCCTGGCCCTAGGCCTCTCAGG------------------GGTGGGTACATCAAGTGAGG 84

Mm_409+ AGCTATCCCTGGGCCTCTCAAATT------------CACCATGAGAGCTTCTCTGCAAGG 94

Zf_467+S2 CTGTGCCTATACTAATATCAAACTTGATGTGATATTTAACCTTTAAAATACAGTGAAAGA 120

* * * * *** * **

Hu_400+ AAGTCACACTGTCAGGGCAGAAAGAGGGGCTGGGTGGGGCCGGACAGGCTCTGC-TGGCT 143

Mm_409+ AAGTCACCCCATCAGGGCAGA--GTGGTTCTAGGTGGGGCTAAACAGGCTCTACCAGGCA 152

Zf_467+S2 TGTACTTATTATTTATATAGATTGTATGTCTCATTAACGCAAACCGTTGTCTACTTCCCT 180

* * *** * ** * ** * *** * *

Hu_400+ CAGGATCCCGGCAGGACAGGCTGCCCAACCCAGCCCCAAAACTCCCATCTTCTGTCCAAC 203

Mm_409+ CAGTATCCCTGCAGGGCAGACTGCCCAGCCTCCTGTTCACG--------------CCCAT 198

Zf_467+S2 A-----------TTGGTTGACTAAGCTACACGTCTTTGCCCGTACACTGTTAGGTATACA 229

* * ** * *

Hu_400+ GCCCAAGCCTGCTTCCTCCCTGCCCC---------------ACCTCACCTCACACTGCCC 248

Mm_409+ GCTTTTTCCTGCCTGCTGCCTGCTCCTCCCCTTCTGGGCCGTGCCAGGCTCATGCAGAGC 258

Zf_467+S2 A-------------ACCAATTAGGTCTCCCGCTCCAGAGTAAGTGGGGGTTGAAATCATC 276

* * * * *

Hu_400+ AGACTG----GTGGGGCTGCATTTTTGG--GGC-CGGTGCCAGGTGTGATGGA-----GG 296

Mm_409+ AGACCA----GTACAGCCGCATTCTTGG--GCAAGTGTCTCTGGTGTGAATGACCCTGGG 312

Zf_467+S2 GGAGCGACGCGTCATATAGTTTCTTACGGAGATAGGGCGCAAAGTGCAACTTCGATT-AA 335

** ** * * * * * * *** *

Hu_400+ ACAGCCCAGGTCAGGAGGTCAGGGCCCCGTTTCCTCCCAAGCCAGGATG-ACTCAGTGAT 355

Mm_409+ GCTGGTCAGGTCAGAAGGTCAGGGCCTTGTTCCCTCCAGTGCCCAAGTG-ATA------- 364

Zf_467+S2 TATGCCGCCCATAGAACATTCA----ACATT-ACTTCGAAGCTGCCCCACCCCACGTGCA 390

* ** * * ** ** * **

Hu_400+ GGACAGACTAGGCCACAAGGGG-CCGAACACAAA---GCCA-AAT--TAGGG-------- 400

Mm_409+ -GATGAGTCAGGCTACAAAGGGGCAGAACAAGAA---ACCA-AAC--CGAGT-------- 409

Zf_467+S2 GGGTC-TTTTGGCGGCGAGAAGGTCAACTACGTTTTAGGTAAGATTTTCTGTGACTGTTG 449

* *** * * * * * * * *

Hu_400+ ----------------- 400

Mm_409+ ----------------- 409

Zf_467+S2 GTCACGCCCCGAGTCCT 466

* 86 count (3-way identical characters)

- 216 count (null characters)

Alignment #20

Hu_400+ TTCTGACACAACCTGCTACACAT----CTTGGATCCCACTTGTCAAGCCTGGCCCTAG-- 54

Mm_409+ CTATCCCACAGACTGGTGACCCTGGAGCTCAGACACTCCACTGGAGAGCTATCCCTGG-- 58

Zf_467+S3 ---AGGAGTAGGCAACTAAAGATGCGTATTTTAGGTTCCTCTTCAGCCCAAACTACACAC 57

* * * * * * * * * *

Hu_400+ --GCCTCTCAGG------GGTGGGTACATCAAGTGAGGAAGTCACACTGTCAGGGCAGAA 106

Mm_409+ --GCCTCTCAAATTCACCATGAGAGCTTCTCTGCAAGGAAGTCACCCCATCAGGGCAGA- 115

Zf_467+S3 CCTAACGTGGGCTTCAGGGATGTCTTTTGCATCTTACCTAATTTACACGGAGCCGCAGTA 117

* * * * ****

Hu_400+ AGAGGGGCTGGGTGGGGCCGGACAGGCTCTGC-TGGCTCAGGATCCCGGCAGGACAGGCT 165

Mm_409+ -GTGGTTCTAGGTGGGGCTAAACAGGCTCTACCAGGCACAGTATCCCTGCAGGGCAGACT 174

Zf_467+S3 ATTAAGTATACTTTTGCAGAAGTAGTCTGCCCATAAATTGAAAGCTGGTCGCAGCTTGTT 177

* * * ** ** * * * * * *

Hu_400+ GCCCAACCCAGC-CCCAAAACTCCCATCTTCTGTCCAA--CGCCCAAGCCTGCTTCCTCC 222

Mm_409+ GCCCAGCCTCCT-GTTCACG--------------CCCA--TGCTTTTTCCTGCCTGCTGC 217

Zf_467+S3 AGCCCAACTCGTATCCAGTCATTGCATCTTGTGACTTTGCAGGCTAAGCTCCCCTCATAA 237

** * * * * * * *

Hu_400+ CTGCCC-C---------------ACCTCACCTCACACTGCCCAGACTGGTGGGGCTG--- 263

Mm_409+ CTGCTC-CTCCCCTTCTGGGCCGTGCCAGGCTCATGCAGAGCAGACCAGTACAGCCG--- 273

Zf_467+S3 GGGGTTTACTCGCTCATCCTATGCGTAATACTCATACATGGGCTTCAAGTTTAGGTAGCC 297

* **** * * ** *

Hu_400+ CATTTTTGGGGC-------------CGGTGCCAGGTGTGATGGA-----GGACAGCCCAG 305

Mm_409+ CATTCTTGGGCA------------AGTGTCTCTGGTGTGAATGACCCTGGGGCTGGTCAG 321

Zf_467+S3 CATTGACGGTAGAATTACCAATTGAATATTGAGGATTAAATATGGCACGGGGACCTAGAC 357

**** ** * * * * ** *

Hu_400+ GTCAGGAGGTCAGGGCCCCGTTTCCTCCCAAGCCAGGATGACTCAGTGATGGACAGACTA 365

Mm_409+ GTCAGAAGGTCAGGGCCTTGTTCCCTCCAGTGCCCAAGTGATA--------GATGAGTCA 373

Zf_467+S3 GTCACAAAGTCTTCGTTAATCTAGGTGTACAATCTTTGTACTCATGTG--GCGTCCGAAA 415

**** * *** * * * * * *

Hu_400+ GGCCACAAGGGG-CCG-----AACACAAAGCCAAATTAGGG----------- 400

Mm_409+ GGCTACAAAGGGGCAG-----AACAAGAAACCAAACCGAGT----------- 409

Zf_467+S3 GGATAAAATGAGGAAATGTTGGTCTTCAACAAACACTTCGTTCATAAGGTTT 467

** * ** * * * ** * * *

* 90 count (3-way identical characters)

- 140 count (null characters)
